# Long-term ultrasound insights into camelid urology: a retrospective analysis of urinary tract disorders in 1,100 dromedary camels (*Camelus dromedarius*) (2012–2025)

**DOI:** 10.3389/fvets.2026.1742804

**Published:** 2026-02-09

**Authors:** Mohamed Tharwat, Hazem M. M. Elmoghazy

**Affiliations:** 1 Department of Clinical Sciences, College of Veterinary Medicine, Qassim University Buraidah, Saudi Arabia; 2 Department of Clinical Medicine, University Veterinary Hospital, Qassim University, Buraidah, Saudi Arabia

**Keywords:** camels, nephrology, retrospective study, ultrasound, urinary tract diseases

## Abstract

**Introduction:**

Urinary tract disorders are common in dromedary camels and present diagnostic challenges due to non-specific clinical signs and limited field diagnostic tools. Ultrasonography is a valuable, non-invasive modality for evaluating renal and lower urinary tract abnormalities. This long-term retrospective study aimed to describe the ultrasonographic findings associated with urinary tract disorders in dromedary camels and to assess the diagnostic utility of ultrasonography as a primary imaging modality.

**Methods:**

Records of 1,100 camels (3 months−20 years) examined between 2012 and 2025 were reviewed. Based on primary clinical presentation and ultrasonographic observations, animals were classified into six descriptive groups: hemorrhagic (G1), inflammatory (G2), infectious (G3), obstructive (G4), traumatic (G5), and neoplastic (G6). Sonographic features were summarized descriptively, and ultrasound-guided aspiration was performed when indicated for pathogen identification.

**Results:**

G1 (*n* = 300) included hemorrhagic conditions characterized by hematuria, bladder clots, thickened or corrugated bladder walls, nephrolithiasis, hydronephrosis, renal abscesses, pyelonephritis, and bladder rupture. G2 (*n* = 332) comprised inflammatory disorders, including cystitis with thickened bladder walls and echogenic luminal deposits, balanitis with pelvic urethral dilation, and bladder paralysis with severe distension and hyperechoic sediment. G3 (*n* = 179) involved infectious diseases, presenting with single or multiple renal abscesses; ultrasound-guided aspiration identified *Staphylococcus lugdunensis* and *Staphylococcus aureus* in sampled cases. G4 (*n* = 214) consisted of obstructive disorders associated with urinary retention, urethral dilation, bladder distension, and renal or bladder calculi producing acoustic shadowing. G5 (*n* = 73) included traumatic conditions such as uroperitoneum and bladder rupture, identified ultrasonographically by free abdominal fluid, floating viscera, and collapsed thick-walled bladders. G6 (*n* = 2) represented neoplastic disease with irregular renal masses of mixed echogenicity.

**Conclusion:**

Ultrasonography provided a detailed assessment of a wide range of urinary tract disorders in dromedary camels and proved to be a practical, non-invasive diagnostic tool in both field and hospital settings. The retrospective data demonstrate its value in identifying lesion location, tissue alterations, and associated structural abnormalities, even when laboratory or advanced imaging data are incomplete. These findings support ultrasonography as a primary diagnostic method for urinary tract evaluation, enhancing clinical recognition and management of urinary disorders in camels.

## 1 Introduction

The dromedary camel (*Camelus dromedarius*) is uniquely adapted to arid environments, with specialized renal physiology that allows for the conservation of water through the production of highly concentrated urine (1–3, 31). While essential for desert survival, this adaptation may predispose camels to renal disorders (4). Thus, urinary tract diseases in dromedaries pose a significant challenge for field veterinarians (5). These renal disorders not only affect camel health and productivity but also have economic implications for pastoral communities reliant on camel resources, highlighting the need for accurate and timely diagnosis (6, 7). Common urinary disorders in dromedaries include pyelonephritis, hydronephrosis, urolithiasis, neoplasia, and renal abscesses (8–13).

The clinical diagnostic approach to renal disorders in camels typically involves case history, physical examination, laboratory tests, and diagnostic imaging techniques (14, 15). However, vague histories, non-specific clinical signs, and inconclusive laboratory findings often limit the accuracy of conventional diagnostic methods in field conditions (13). Among imaging modalities, sonography has proven particularly valuable as a non-invasive tool for the early detection of renal diseases in dromedary camels (16). This technique enables accurate identification of kidney conditions, including pyelonephritis and renal abscesses, thereby supporting timely intervention and treatment monitoring when necessary (13).

Ultrasonography has become a vital diagnostic tool in dromedary camel medicine, particularly for evaluating gastrointestinal, cardiopulmonary, and hepatobiliary conditions (6, 7, 17–25). Its application in renal evaluation has enabled the detection of nephrolithiasis, hydronephrosis, pyelonephritis, and renal abscessation (13). Despite the increasing use of ultrasonography, there remains a lack of large-scale, long-term studies systematically describing the spectrum, prevalence, and sonographic characteristics of urinary tract disorders in dromedary camels, limiting evidence-based clinical decision-making and standardized management protocols (17).

Existing literature on urinary tract disorders in dromedary camels is largely limited to case reports and cross-sectional studies, which provide fragmented insights into disease presentation and progression (13). Accordingly, a comprehensive long-term evaluation integrating clinical and ultrasonographic findings is needed. The present study addresses this gap by retrospectively analyzing urinary tract disorders in 1,100 dromedary camels over a 14-year period, using ultrasonography as the primary diagnostic tool. By systematically documenting sonographic features alongside clinical presentations, this research aims to enhance understanding of camelid urology and support improved prevention, diagnosis, and management of urinary tract diseases in dromedary camels.

## 2 Materials and methods

### 2.1 Study design and population

This retrospective observational study reviewed medical records from the University Veterinary Hospital at Qassim University, Saudi Arabia, covering the period from 2012 to 2025. A total of 27,337 clinically affected dromedary camels (*Camelus dromedarius*) were examined during this period. Cases were categorized into reproductive conditions (14,402 camels), surgical problems (6,825 camels), and medical illnesses (6,110 camels). Among the medically diagnosed animals, all camels with documented urinary tract disorders were included, resulting in 1,100 camels (18%). No additional inclusion or exclusion criteria were applied beyond documentation of urinary tract disorders, reflecting the retrospective nature of the dataset.

### 2.2 Clinical evaluation and handling

Each camel underwent a comprehensive clinical examination, including assessment of respiratory rate, heart rate, rectal temperature, mucous membrane condition, and auscultation of thoracic and gastrointestinal regions. Routine handling and restraint were performed by trained veterinary personnel, ensuring animal welfare and staff safety. For ultrasound-guided biopsies or aspirations, sedation was achieved with intravenous xylazine (2%, 0.2 mg/kg BW), and local anesthesia was administered using 10 ml of 2% procaine hydrochloride. All procedures were part of routine clinical care. Ethical approval for retrospective analysis was granted by the *Ethics Committee for Animal Use* at Qassim University (Buraydah, Saudi Arabia), following the *Guide for the Care and Use of Agricultural Animals in Research and Teaching* (26). No specific license or protocol number was assigned due to the retrospective study design.

### 2.3 Classification and diagnostic criteria

Affected camels were classified into six clinical categories based primarily on clinical presentation and ultrasonographic observations, with supplementary information from clinical history and laboratory or histopathological confirmation when available. Because this study is retrospective, not all cases had confirmatory tests; terminology reflects the level of diagnostic certainty for each category. Table 1 summarizes the diagnostic category, diagnostic basis, confirmatory tests, and notes on limitations for each group.

**Table 1:** Diagnostic basis for each clinical category of urinary tract disorders in dromedary camels (*n* = 1,100).

**Diagnostic category**	**Diagnostic basis**	**Confirmatory test**	**Notes**
**G1** – Hemorrhagic conditions (*n* = 300)	Ultrasound ± clinical signs	Limited urinalysis	Echogenic bladder contents, blood clots, thickened bladder wall; hematuria confirmed in subset
**G2** – Inflammatory/possible cystitis (*n* = 332)	Ultrasound ± clinical signs	Limited urinalysis/culture	Bladder wall thickening, corrugation, hyperechoic deposits; etiology (infectious vs. sterile) not confirmed in most cases
**G3** – Cavitary/cystic renal lesions (*n* = 179)	Ultrasound ± aspiration when available	Culture-confirmed Staphylococcus spp.	Lesions suggestive of abscess; most classified by sonography alone
**G4** – Obstructive conditions (*n* = 214)	Ultrasound ± clinical signs	Rare laboratory confirmation	Distended bladder, urethral dilation, calculi with acoustic shadowing
**G5** – Traumatic conditions (*n* = 73)	Ultrasound ± clinical history	Not available	Free abdominal fluid, floating viscera, collapsed thick-walled bladder
**G6** – Neoplastic conditions (*n* = 2)	Ultrasound + histopathology	Histopathology confirmed	Renal mass, tumor type specified

#### 2.3.1 Group 1: hemorrhagic conditions (G1)

Camels presenting with gross hematuria and echogenic intraluminal material, including blood clots, identified via ultrasound. Some cases also showed bladder wall thickening or renal structural changes. Although some cases had laboratory confirmation via urinalysis, the primary basis for classification was sonographic and clinical presentation, reflecting the study focus on initial clinical appearance.

#### 2.3.2 Group 2: inflammatory/possible cystitis (G2)

Camels with sonographic evidence of bladder wall thickening, corrugation, and hyperechoic intraluminal deposits, often accompanied by dysuria or anuria. While suggestive of cystitis, infectious or sterile origin could not be confirmed in most cases due to incomplete urinalysis or culture data. Descriptions of anuria or dysuria have been consolidated to avoid repetition.

#### 2.3.3 Group 3: cavitary/cystic renal lesions (G3)

Camels with single or multiple renal cavitary or cystic lesions consistent with abscesses. When available, ultrasound-guided aspiration allowed bacteriological confirmation (Staphylococcus spp.). Most cases were classified based on sonographic appearance alone.

#### 2.3.4 Group 4: obstructive conditions (G4)

Camels with urinary retention, bladder distension, urethral dilation, or echogenic calculi with distal acoustic shadowing. Diagnosis relied primarily on ultrasonographic features, supported by clinical signs without repeated mention of anuria or bladder distension.

#### 2.3.5 Group 5: traumatic conditions (G5)

Camels presenting with free abdominal fluid, floating viscera, or collapsed thick-walled bladders, consistent with uroperitoneum or bladder rupture. Classification was based on ultrasound, with clinical history of trauma incorporated without redundant phrasing regarding the number of affected camels.

#### 2.3.6 Group 6: neoplastic conditions (G6)

Camels with renal masses visualized on ultrasound. Histopathological confirmation was available in both cases, specifying the tumor types (renal adenocarcinomas).

This classification system enabled systematic documentation of clinical and ultrasonographic features without statistical comparison between groups, consistent with the descriptive aim of the study.

### 2.4 Sonographic evaluation

All camels underwent ultrasonographic examination of the urinary tract using a SonoScape ultrasound machine (Sonoscape Medical Corp., China) with sector probes (3.5 MHz) and linear transducers (5.0 and 7.5 MHz). Standard clinical settings for depth, gain, and focus were applied consistently throughout the study period. The right kidney was visualized transcutaneously through the upper right flank, and the left kidney was imaged either transrectally or from the caudal left flank. The urinary bladder was examined transrectally. Sonographic findings—including kidney and bladder morphology, echogenic deposits, blood clots, abscesses, uroliths, or masses—were recorded systematically in the medical records. Due to the retrospective design, ultrasonographic images were not uniformly archived for re-evaluation.

### 2.5 Ultrasound-guided procedures

When indicated, lesions were biopsied or aspirated under ultrasound guidance using a 14G × 170 mm spinal biopsy needle after aseptic preparation. Samples were immediately processed for bacteriological and histopathological investigations, depending on clinical indication and lesion accessibility.

### 2.6 Laboratory and treatment data

Prior treatments, when documented, typically included antibiotics, anti-inflammatory agents, multivitamins, appetite enhancers, and intravenous fluids. Laboratory investigations—including urinalysis, urine culture, and hematological/biochemical parameters—were inconsistently recorded, limiting correlation with sonographic findings, disease severity, or treatment outcomes; this limitation is discussed further in Section 4.

### 2.7 Statistical analysis

Due to the retrospective and descriptive nature of this study, formal inferential statistical analyses were not performed. Data are presented as descriptive statistics, including counts, percentages, and distributions of sonographic findings across the six clinical categories, reflecting the primary objective of documenting ultrasonographic and clinical features rather than testing hypotheses or comparing treatments.

## 3 Results

### 3.1 Group 1 (G1 – disorders presenting with grossly discolored urine and intraluminal echogenic bladder content)

**Figure 1:**
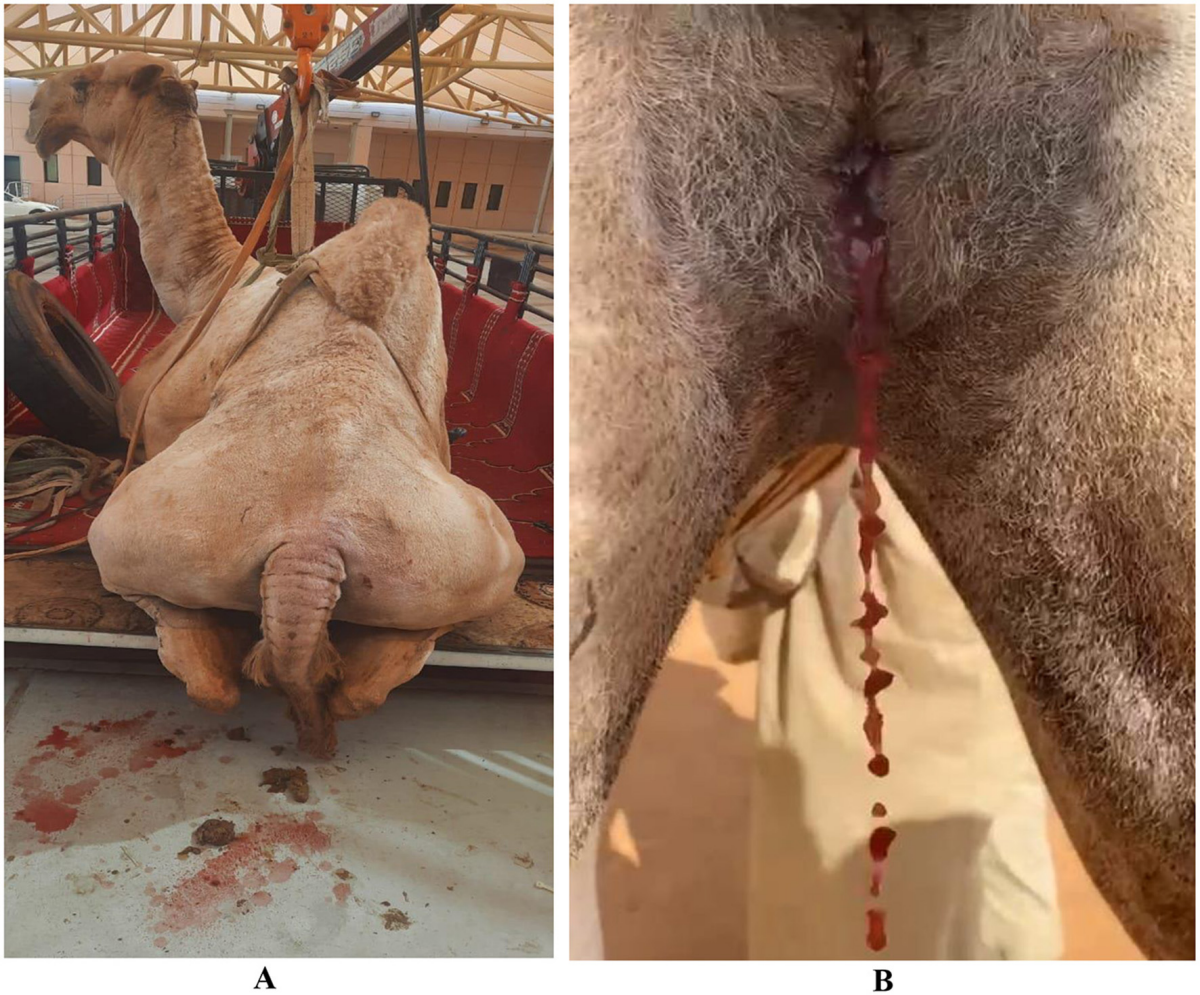
Urine discoloration in a male **(A)** and a female **(B)** dromedary camel with a period of 3 and 6 weeks, respectively.

A total of 300 dromedary camels [225 females (75%), 75 males (25%)], aged between 6 months and 20 years, were presented with a primary clinical complaint of visibly discolored (reddish to brownish) urine lasting from 2 days to 1 year (Figure 1). Additional clinical signs included weakness, urine dribbling, painful urination, and abdominal pain. Case assignment to this group was based primarily on clinical observation of urine discoloration and ultrasonographic findings. Systematic urinalysis was not available to differentiate hematuria from pigmenturia or other causes of urine discoloration. Therefore, the term “hemorrhagic” is used descriptively rather than as a definitive etiologic diagnosis.

Ultrasonographic examination revealed variable intraluminal echogenic material within the urinary bladder, irregular or thickened bladder walls, marked bladder distension, and echogenic urine within the renal pelvis. Additional sonographic findings included renal cavitary or heterogeneous parenchymal lesions consistent with abscessation, renal pelvic dilation consistent with hydronephrosis, echogenic foci with distal acoustic shadowing consistent with nephrolithiasis, collapsed bladder contours with surrounding free abdominal fluid consistent with bladder rupture, and hypoechoic peritoneal fluid with floating abdominal viscera consistent with uroperitoneum.

**Figure 2:**
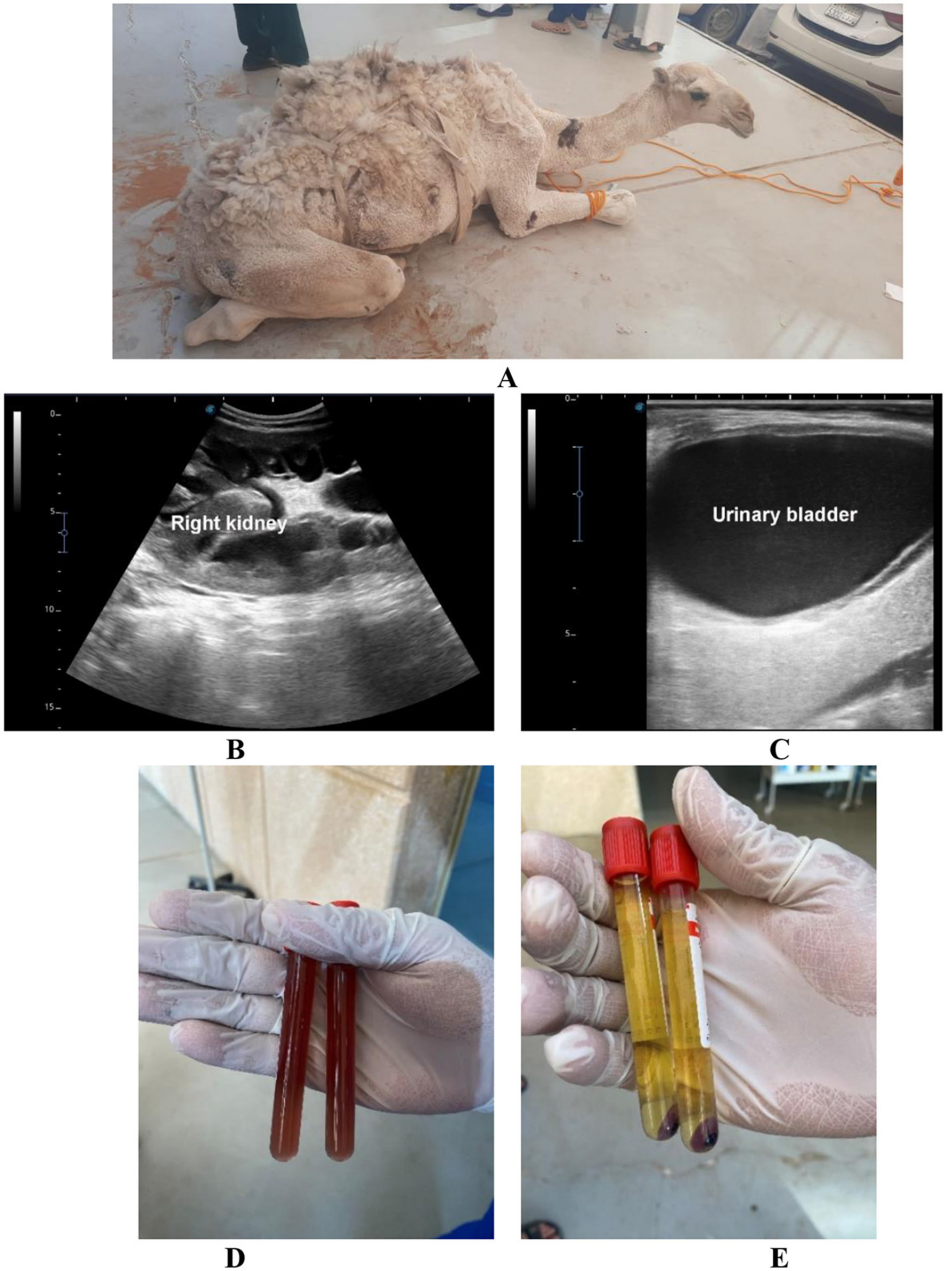
Voiding of red urine in a male dromedary camel calf due to partial urethral obstruction. The animal had a 5-day history of anorexia, abdominal pain, and hematuria **(A)**. Renal ultrasonography was performed using a 3.5 MHz sector probe via right and left flank approaches: bilateral hydronephrosis is visible, with dilated renal pelvises and thinned renal parenchyma **(B)**. Transabdominal ultrasonography of the urinary bladder (linear 5 MHz probe, ventral midline approach) revealed a markedly distended bladder containing echogenic urine, indicating blood clots and hematuria **(C)**. The collected urine appeared deep red **(D)** and, after centrifugation, became light yellow with a red precipitate at the bottom, confirming hematuria **(E)**.

In several cases, overlapping sonographic features involving the kidneys and bladder were observed, highlighting the multifactorial nature of disorders presenting with urine discoloration. Bilateral renal pelvic dilation with a distended urinary bladder containing echogenic urine was documented in a subset of cases (Figure 2). No histopathological or laboratory confirmation of hemorrhage was available for this group.

### 3.2 Group 2 (G2 – disorders characterized by bladder wall thickening, echogenic intraluminal material, or impaired bladder emptying)

This group included 332 camels, subdivided according to predominant sonographic and clinical presentation rather than confirmed etiologic diagnosis.

#### 3.2.1 Camels with bladder wall thickening and intraluminal echogenic material (n = 208)

**Figure 3:**
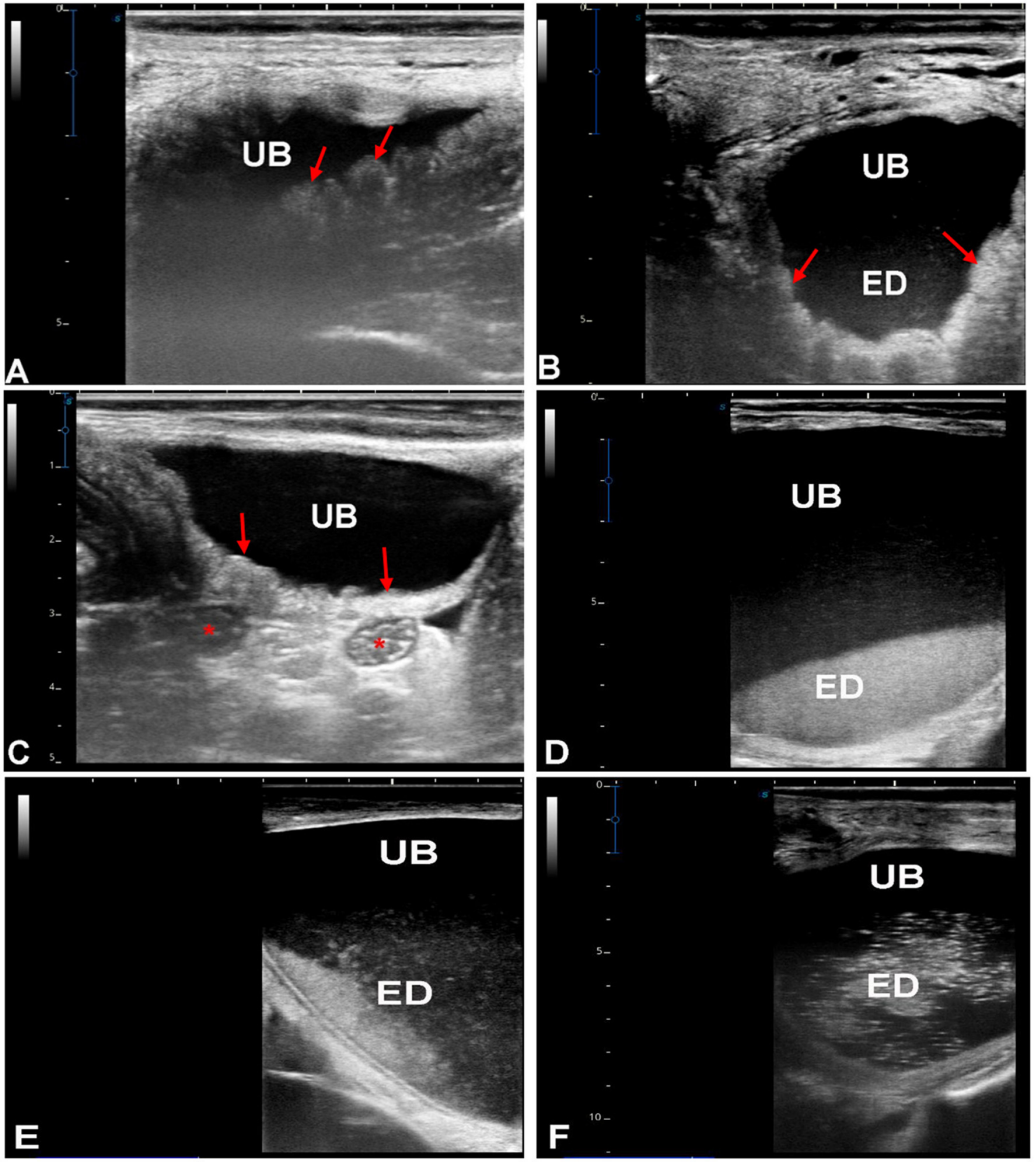
Inflammation of the urinary bladder (UB) in dromedary camels presenting with dysuria and hematuria. Transrectal ultrasonography was performed using a 5 MHz linear probe, oriented longitudinally along the ventral midline. The urinary bladder wall appears thickened and corrugated (arrows; images **A**, **B**, **C**). Adjacent lymph nodes are enlarged (stars; image **C**). Echogenic deposits (ED), representing sediment or inflammatory debris, are visible as hyperechogenic foci in the ventral portion of the bladder **(D, E)**. In severe cases, these deposits may be homogeneously distributed throughout the bladder lumen **(F)**, reflecting extensive inflammation or sediment accumulation.

These camels [150 females (72.1%), 58 males (27.9%), aged 1–18 years] were assigned to this subgroup based on transrectal ultrasonographic identification of a thickened, corrugated urinary bladder wall with variable echogenic to hyperechogenic intraluminal material. Enlargement of adjacent lymph nodes was also observed in some cases. In the absence of urinalysis or urine culture, these findings were considered sonographically consistent with an inflammatory or inflammatory–infectious bladder process, but no distinction between sterile inflammation, infection, crystalline sediment, or fine calculi could be made retrospectively (Figure 3).

#### 3.2.2 Camels with balanitis-associated lower urinary tract dysfunction (n = 70)

**Figure 4:**
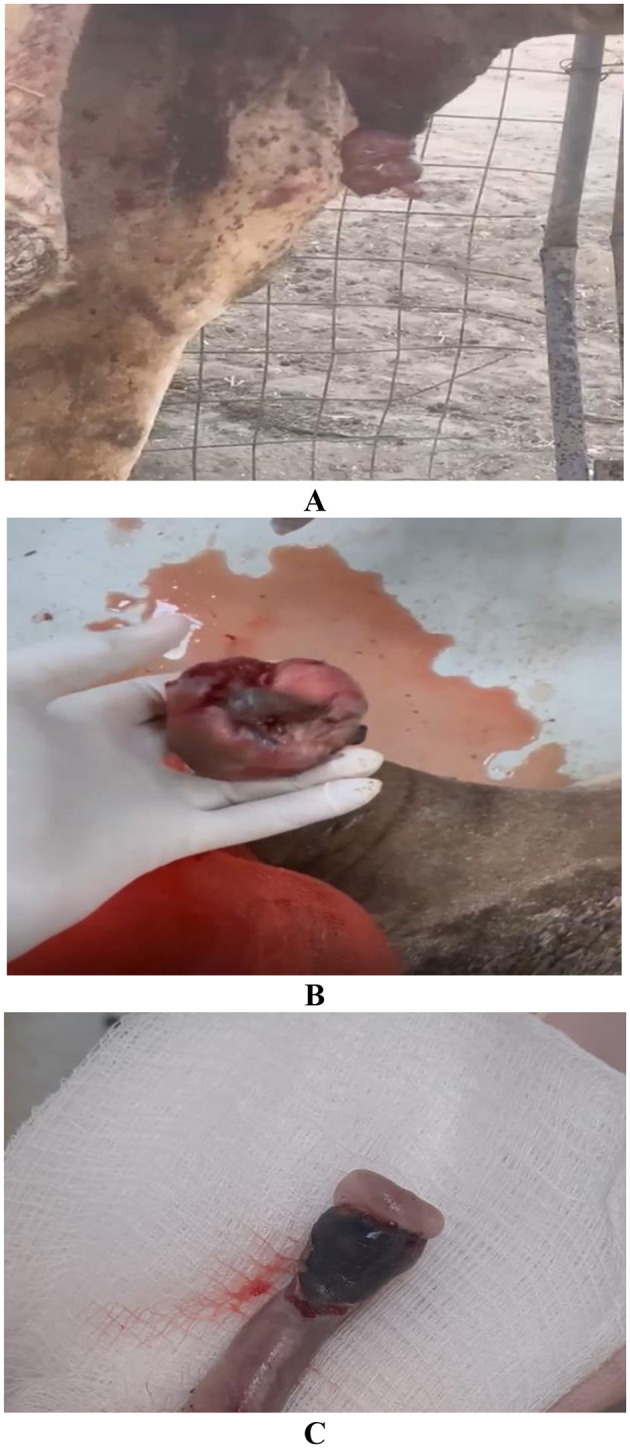
Balanitis in 3 male dromedaries. Animals **(A, B)** exhibited a history of anuria, inability to protrude the penis beyond the prepuce, preputial swelling, rubbing against rough surfaces (suggestive of pruritus), urine dribbling, and dysuria. The penis shown in image **(C)** is for male dromedary had a 1-week history of red-tinged urine dribbling. Clinical examination of all three animals revealed an enlarged, inflamed, and congested glans penis that was painful on palpation.

Seventy male camels aged 5–15 years were admitted with clinical signs including anuria, urine dribbling, inability to protrude the penis, preputial swelling, and painful urination lasting 3–7 days. Clinical examination revealed an enlarged, inflamed, and painful glans penis (Figure 4). Ultrasonography demonstrated a distended urinary bladder and moderate dilation of the pelvic urethra, findings interpreted as functional or partial outflow obstruction secondary to penile and preputial pathology rather than primary urinary tract disease.

#### 3.2.3 Camels with impaired bladder emptying (n = 54)

Fifty-four camels [25 females (46.3%), 29 males (53.7%), aged 5–20 years] presented with prolonged urine dribbling (5–30 days). Ultrasonographic examination revealed an intact but markedly distended urinary bladder containing hyperechoic intraluminal material, with no evidence of rupture or mechanical obstruction. These cases were classified based on sonographic evidence of bladder atony or impaired contractility, without laboratory confirmation of underlying neurologic or inflammatory causes.

### 3.3 Group 3 (G3 – renal cavitary or heterogeneous lesions with microbiological confirmation when available)

**Figure 5:**
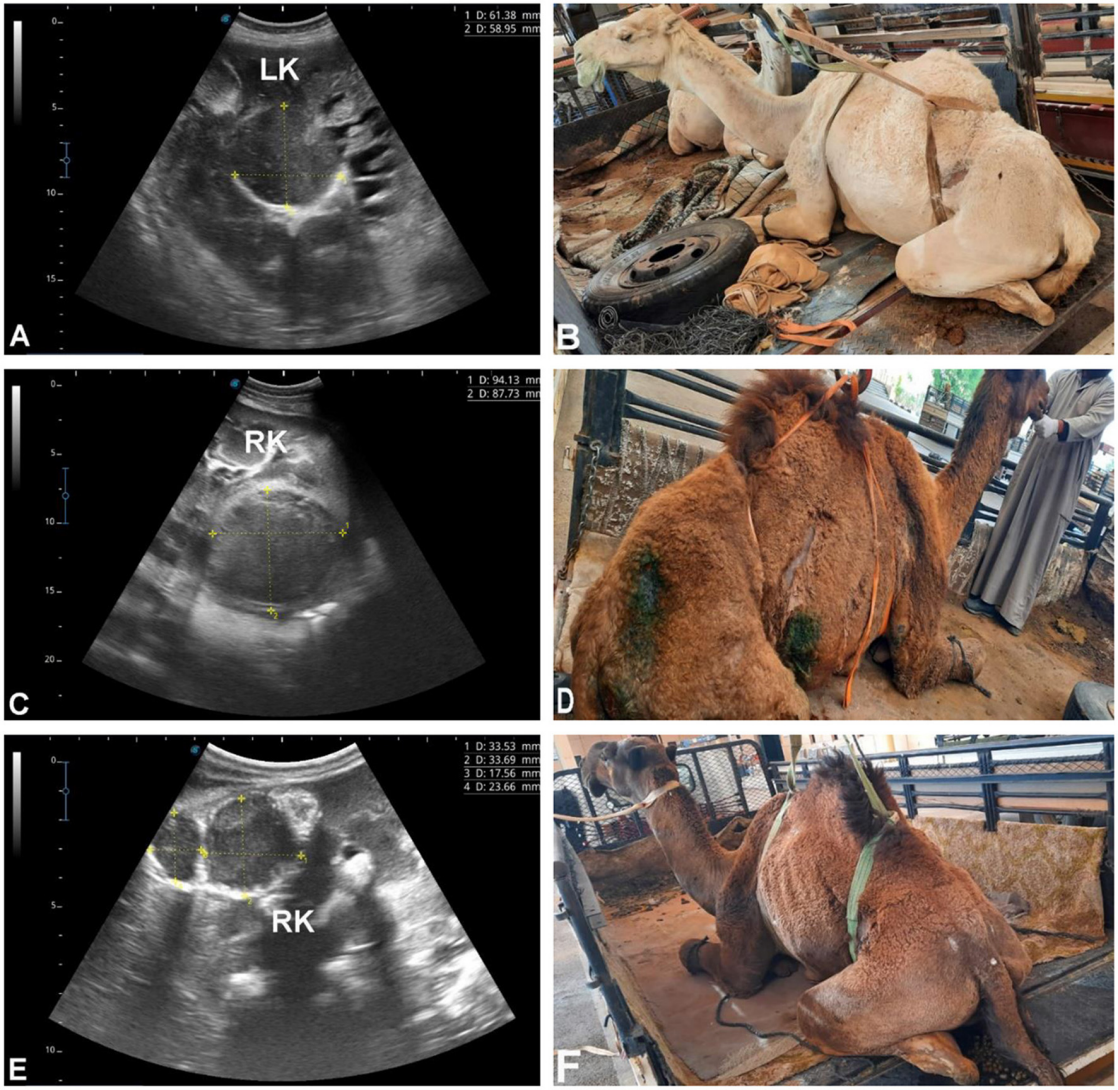
Renal abscesses in dromedary camels. Transabdominal ultrasonography was performed using a 5 MHz sector probe, oriented longitudinally along the flank. **(A)** Shows a hypoechoic abscess in the left kidney (LK) of a female camel presented with chronic weight loss and anorexia **(B)**. **(C)** demonstrates a hypoechoic abscess in the right kidney (RK) of a female camel with multiple, non-resolving skin abscesses **(D)**. **(E)** Depicts multiple hypoechoic and heterogeneous abscesses in the right kidney (RK) of a male camel admitted for weight loss and anorexia **(F)**. The abscesses compress the surrounding renal parenchyma, showing variable echotexture and occasional internal septations.

**Figure 6:**
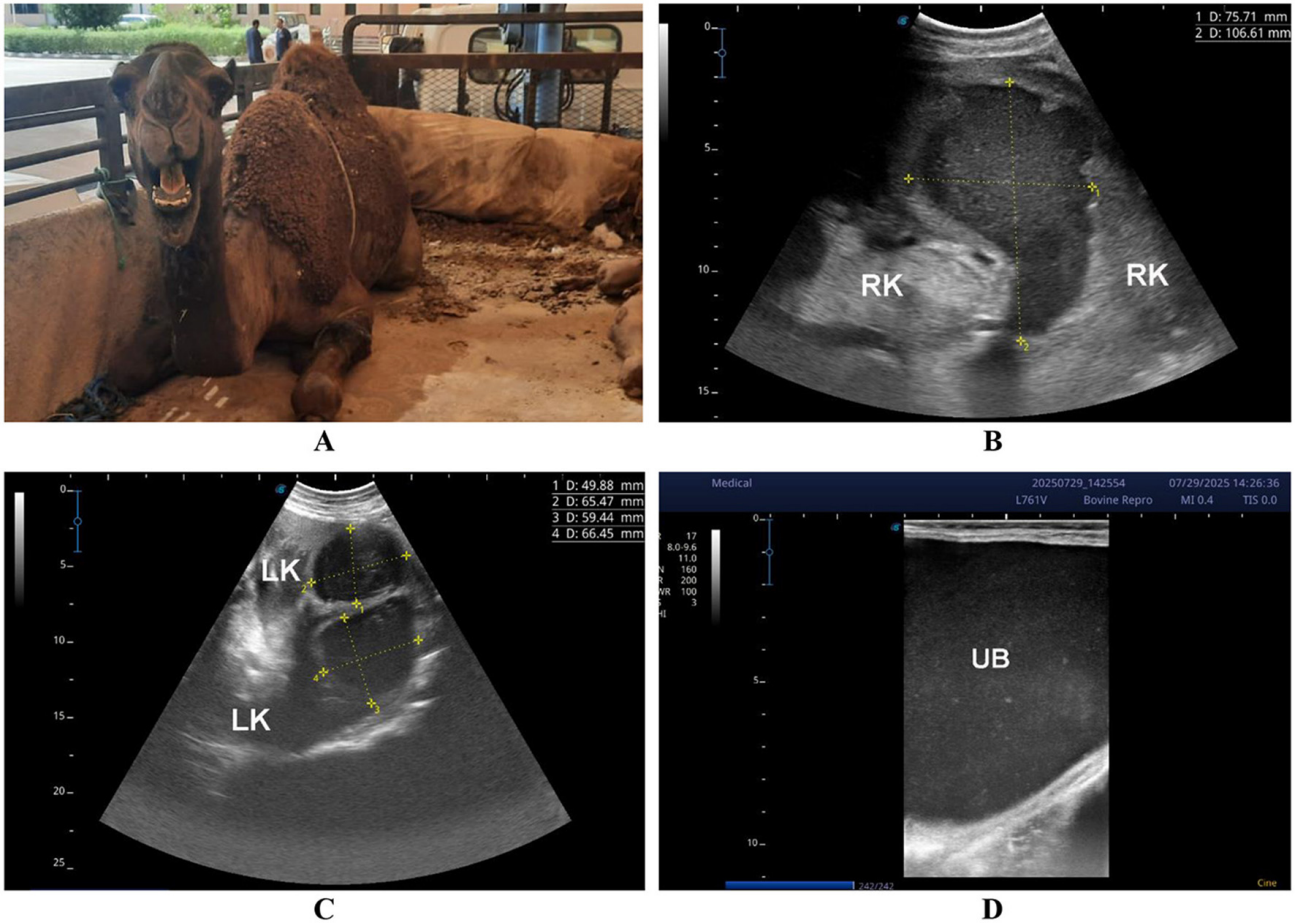
Bilateral renal abscesses in a female dromedary camel. Transabdominal ultrasonography was performed using a 5 MHz sector probe, oriented longitudinally along both flanks. The animal presented with a 20-day history of hematuria **(A)**. **(B)** shows a large hypoechoic abscess occupying the right kidney (RK) parenchyma, while two medium-sized hypoechoic abscesses are visible within the left kidney (LK) parenchyma **(C)**. The urinary bladder (UB) appears distended and contains echogenic urine deposits, indicative of hematuria or debris **(D)**.

**Figure 7:**
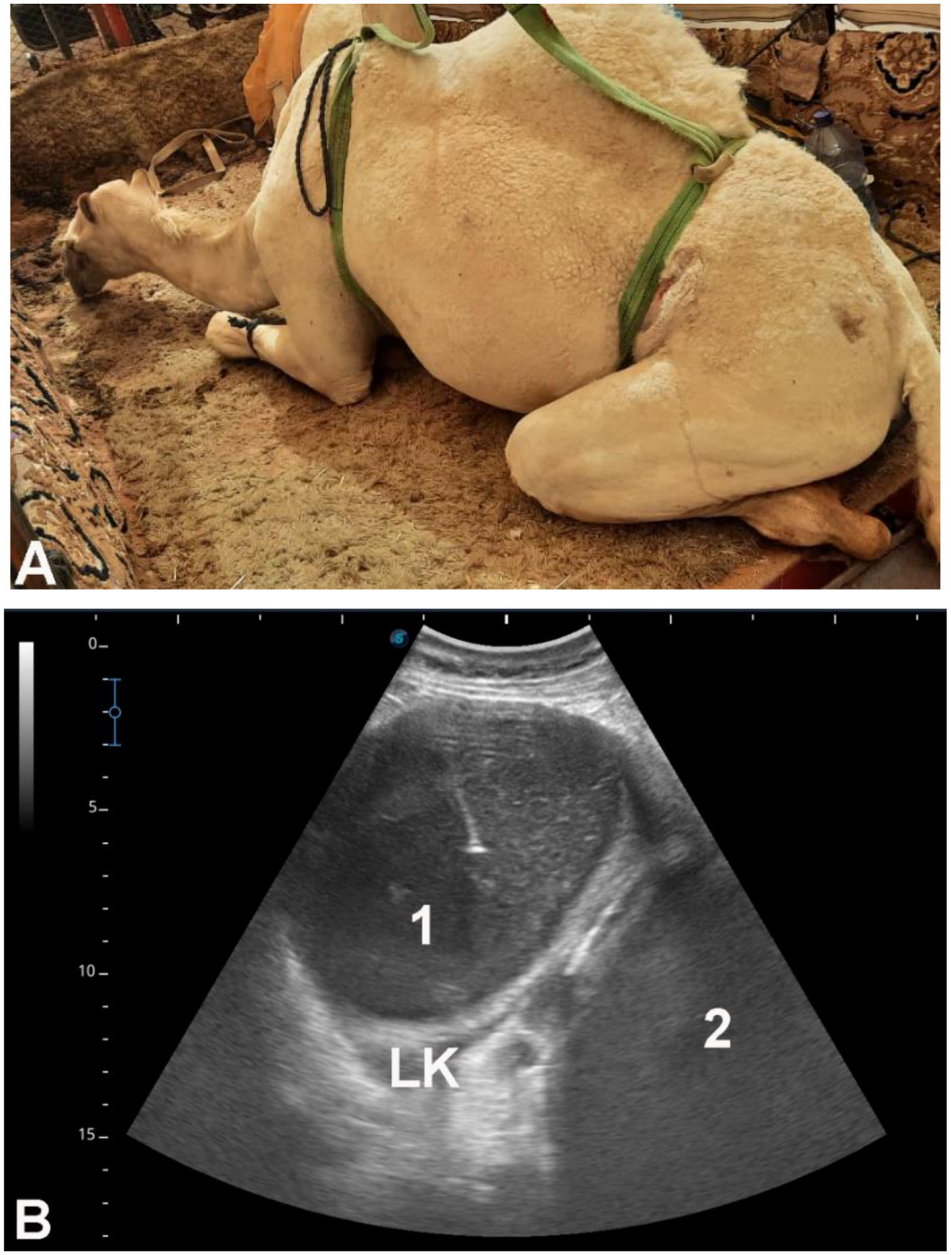
Ultrasound-guided aspiration of renal abscesses in a female dromedary camel. Transabdominal ultrasonography was performed using a 5 MHz sector probe, oriented longitudinally along the left flank. The animal presented with anorexia and depression **(A)**. **(B)** Shows two abscesses within the left kidney (LK) parenchyma (1 = abscess no. 1, 2 = abscess no. 2). A 14G spinal biopsy needle was positioned perpendicular to the transducer (arrow) and inserted into the center of the larger abscess under real-time ultrasound guidance. Pus was successfully aspirated for bacteriological analysis. The surrounding renal parenchyma appears compressed and hyperechoic, consistent with abscess formation.

A total of 179 camels [145 females (81%), 34 males (19%), aged 6–17 years] presented with non-specific systemic signs including inappetence, weight loss, abdominal pain, weakness, constipation, lameness, and, in some cases, urine discoloration. Ultrasonographic examination revealed single or multiple cavitary or heterogeneous renal parenchymal lesions compressing adjacent tissue, located in the left kidney (*n* = 103, 57.5%), right kidney (*n* = 52, 29.1%), or bilaterally (*n* = 24, 13.4%). Lesions varied in echogenicity (hypoechoic, hyperechoic, mixed, or heterogeneous), consistent with suppurative or necrotic processes (Figures 5, 6). Ultrasound-guided aspiration was performed in a subset of cases where lesions were accessible (Figure 7). In these cases, microbiological culture yielded Gram-positive cocci arranged in grape-like clusters, with biochemical identification confirming *Staphylococcus lugdunensis* and *Staphylococcus aureus*. Cases lacking aspiration or culture were classified based on sonographic appearance alone and are therefore described as lesions consistent with renal abscessation rather than definitively confirmed abscesses.

### 3.4 Group 4 (G4 – obstructive disorders based on sonographic evidence of urinary outflow obstruction)

**Figure 8:**
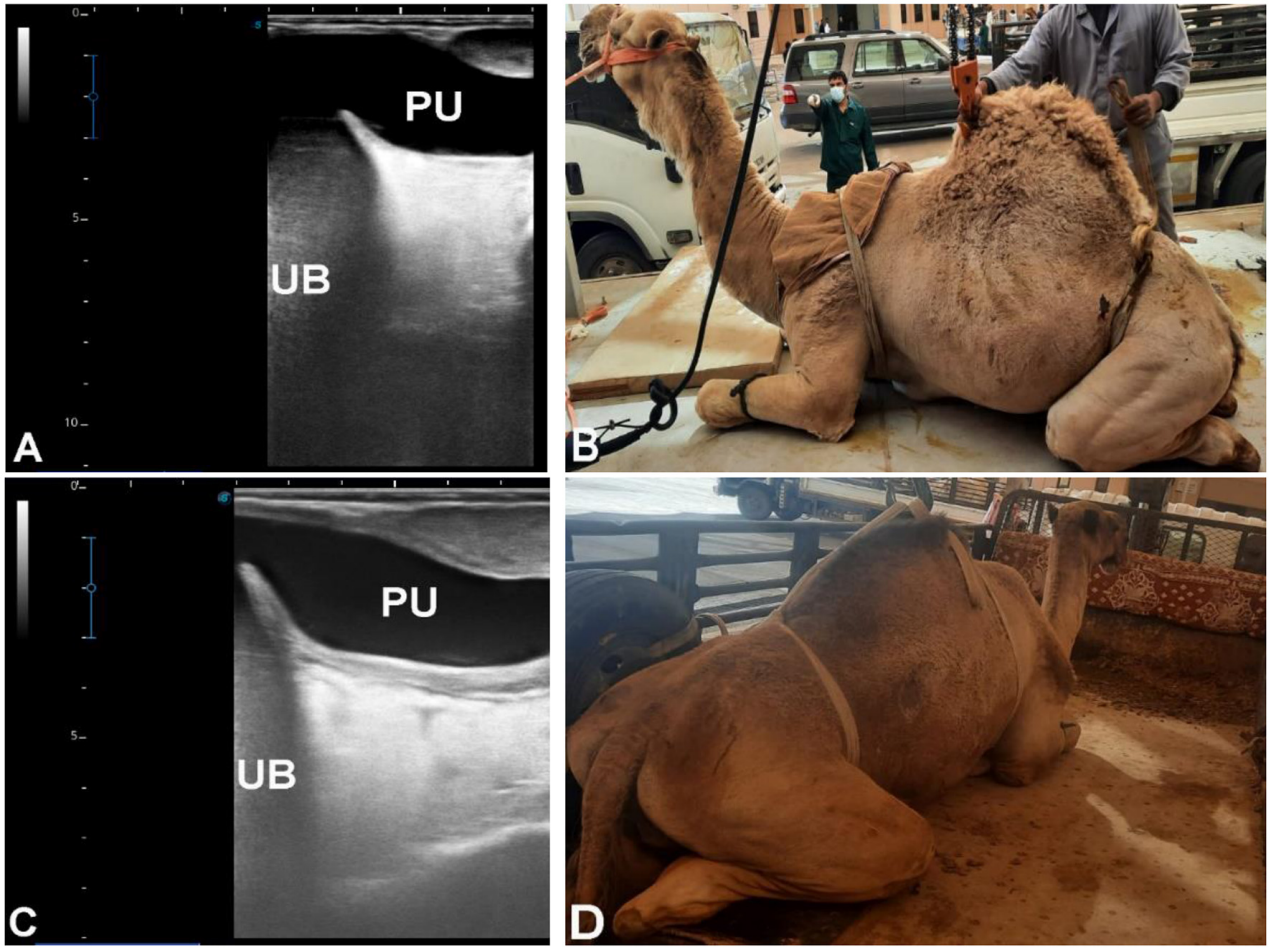
Urine retention in male dromedary camels. Transrectal ultrasonography was performed using a 5 MHz linear probe, oriented longitudinally along the pelvic floor (**A** and **C**). Both animals presented with a history of urine retention for 5 days **(B)** and 7 days **(D)**, with elevated blood urea nitrogen (88 and 178 mg/dl) and serum creatinine (15.9 and 25 mg/dl) levels. Ultrasonography revealed a markedly distended urinary bladder (UB) and a significantly dilated pelvic urethra (PU). The bladder wall appears smooth but stretched, and urine is hypoechoic, consistent with retention due to obstruction.

**Figure 9:**
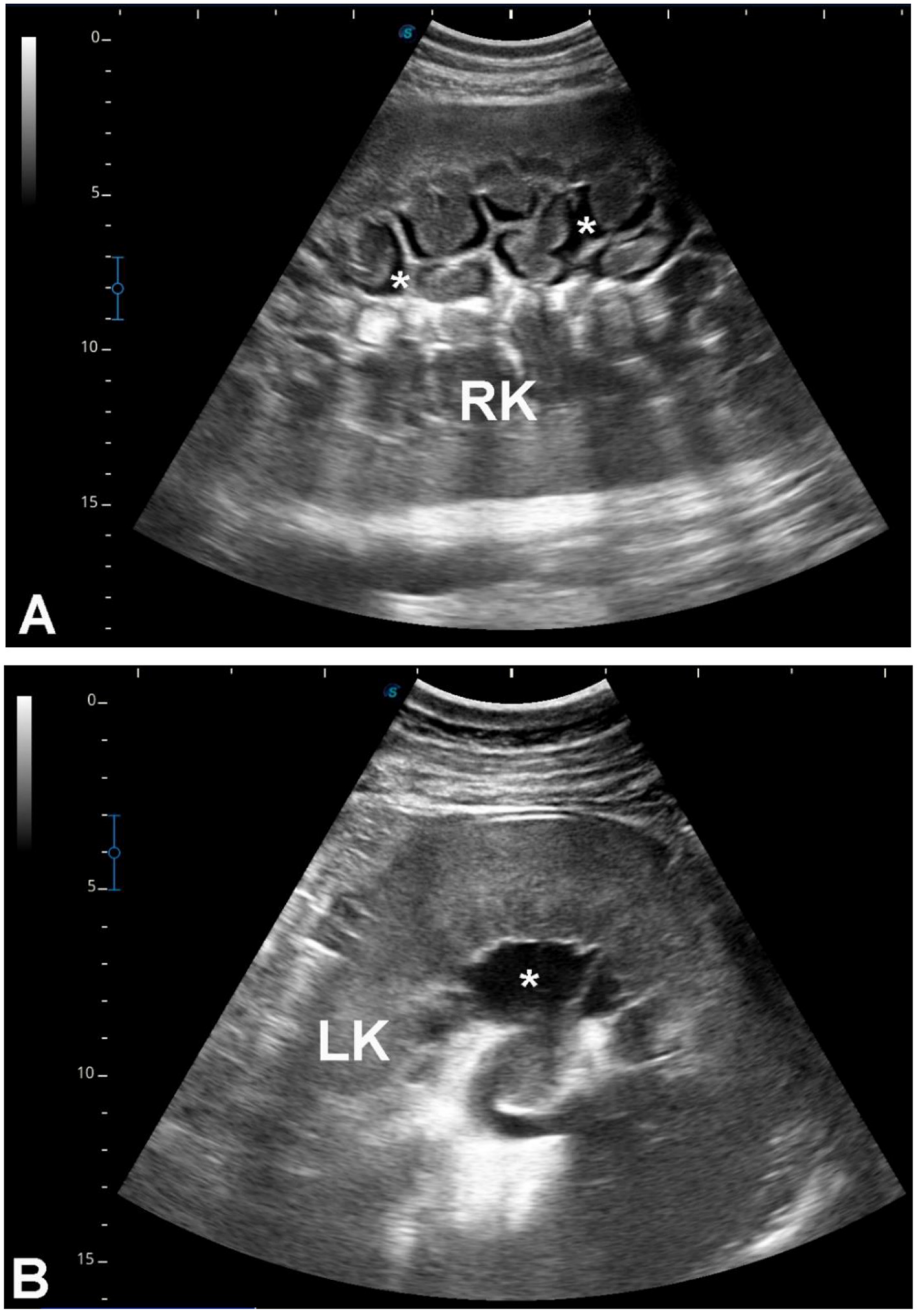
Renal ultrasonography in male dromedary camels with urine retention. Sector probe (3.5 MHz) was used for imaging. **(A)** Right kidney (RK) visualized from the cranial right paralumbar fossa with longitudinal orientation, showing mild dilatation of the renal sinus (star). **(B)** Left kidney (LK) imaged from the caudal left paralumbar fossa in longitudinal orientation, demonstrating moderate renal sinus dilatation (star). These findings are consistent with urinary obstruction and compensatory changes in the kidneys secondary to urine retention.

**Figure 10:**
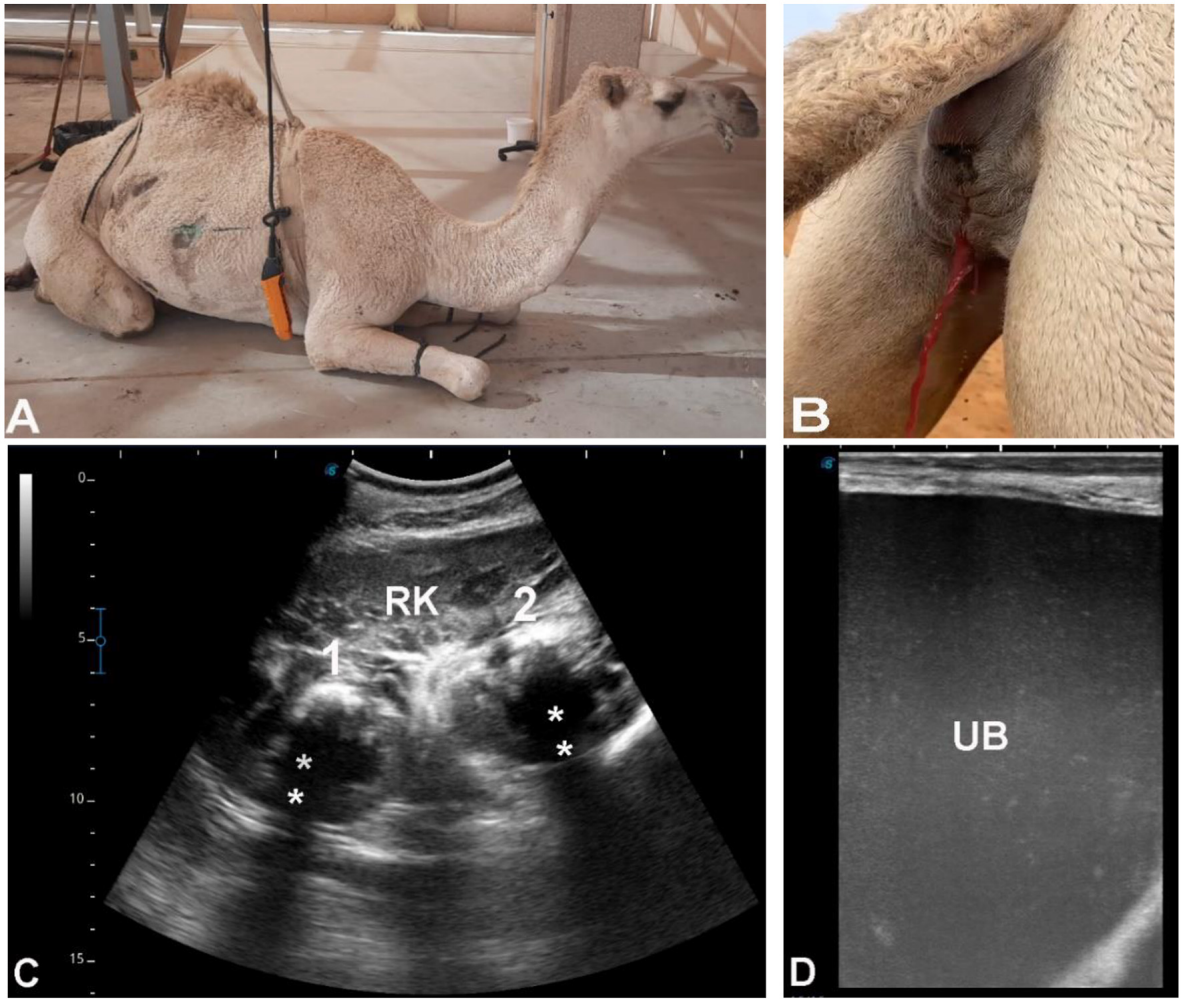
Ultrasonographic findings in a female dromedary camel with a 3-month history of hematuria. **(A)** Clinical observation of bloody urine. **(B)** Urine after spontaneous voiding showing complete discoloration. **(C)** Renal ultrasonography of the right kidney (RK) using a 3.5 MHz sector probe in longitudinal orientation, showing two nephroliths (1 & 2) with distal acoustic shadowing (stars), consistent with nephrolithiasis. **(D)** Transrectal ultrasonography of the urinary bladder (UB) in longitudinal orientation, demonstrating homogeneously distributed hyperechoic deposits throughout the bladder lumen, indicative of cystic calculi or sediment accumulation.

**Figure 11:**
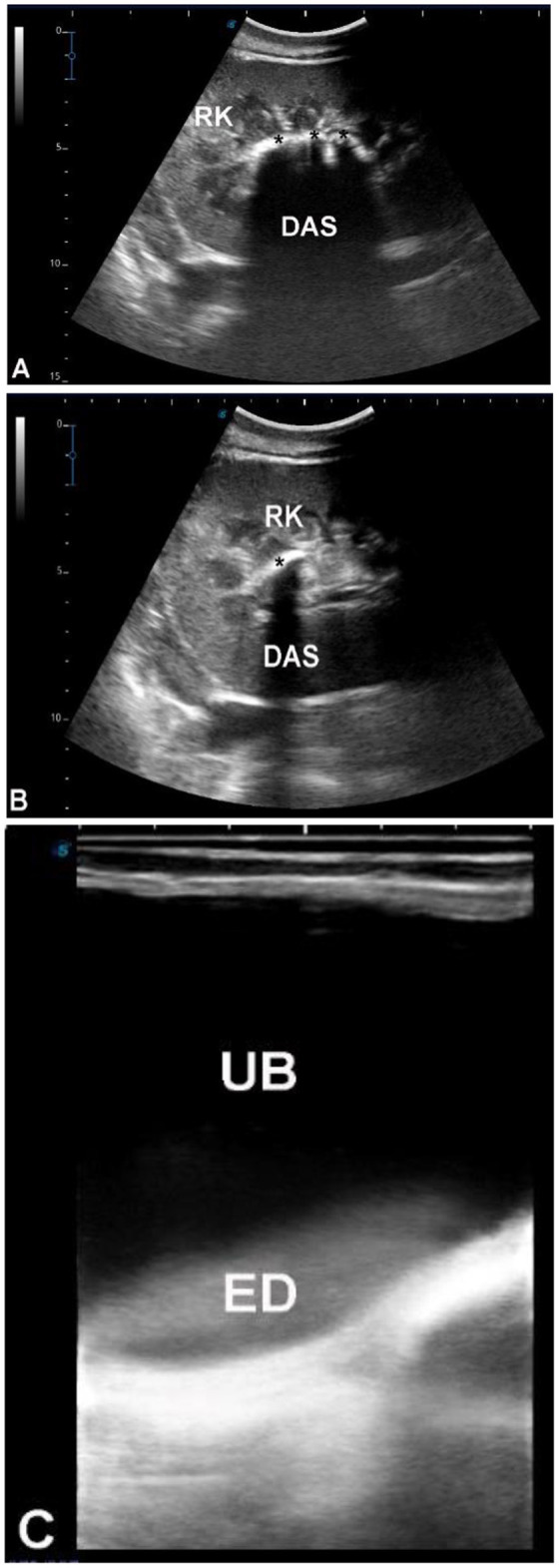
Ultrasonographic findings in a female dromedary camel with a 2-month history of decreased appetite, polyuria, and dysuria. **(A)** Longitudinal ultrasonography of the right kidney (RK) using a 3.5 MHz sector probe, showing multiple renal calculi (black stars). **(B)** Close-up longitudinal view of the RK, demonstrating an additional solitary calculus (black star). **(C)** Transrectal ultrasonography of the urinary bladder (UB) in longitudinal orientation, revealing echogenic deposits (ED) within the bladder lumen with distal acoustic shadowing (DAS), consistent with bladder calculi.

This group included 214 camels, classified based on clear ultrasonographic evidence of urinary tract obstruction, a condition for which ultrasound is considered diagnostically reliable. Camels with urine retention (*n* = 119) [7 females (5.9%), 112 males (94.1%), aged 2–14 years] showed marked bladder distension and dilation of the pelvic urethra (Figure 8), with mild dilation of the renal sinus consistent with secondary back-pressure changes (Figure 9). An additional 95 camels [22 females (23.2%), 73 males (76.8%), aged 3–20 years] presented with anuria, urine dribbling, abdominal pain, and distension. Ultrasonography demonstrated echogenic foci producing distal acoustic shadowing consistent with renal calculi, along with echogenic intraluminal bladder material (Figures 10, 11). Given the characteristic acoustic shadowing and anatomical location, these findings were considered diagnostic of obstructive urolithiasis.

### 3.5 Group 5 (G5 – traumatic and urine leakage–associated disorders)

**Figure 12:**
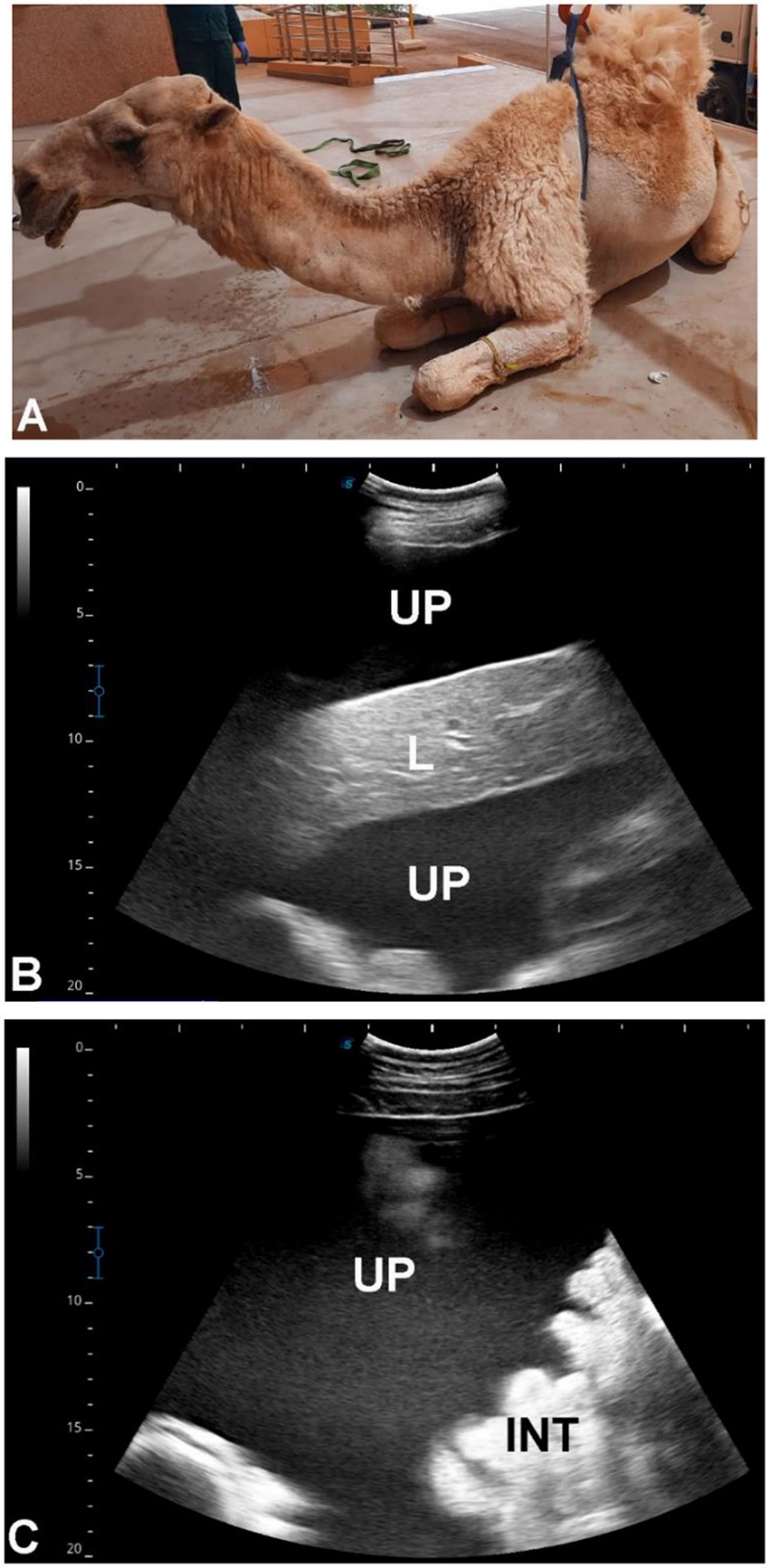
Ultrasonographic findings in a male dromedary camel with a ruptured urinary bladder. The animal presented with anuria for 20 days and depression **(A)**; blood urea nitrogen was 235 mg/dl and serum creatinine >30 mg/dl. **(B)** Longitudinal abdominal ultrasonography using a 3.5 MHz sector probe showing massive uroperitoneum (UP) with the liver (L) floating within anechoic fluid. **(C)** Transverse abdominal ultrasonography revealing floating intestinal loops (INT) suspended in the free peritoneal fluid, consistent with uroperitoneum secondary to bladder rupture.

**Figure 13:**
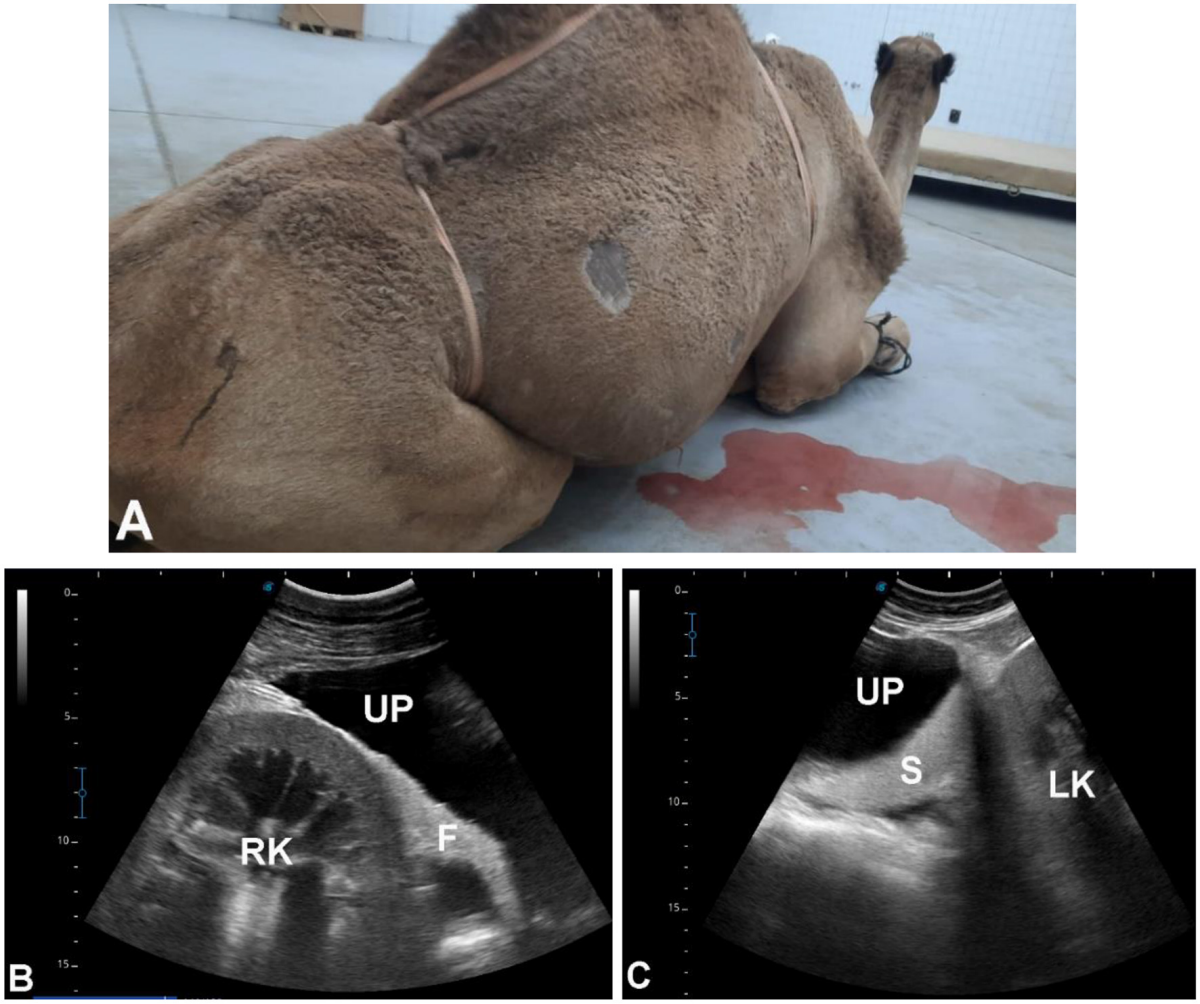
Ultrasonographic evaluation of a male dromedary camel with ruptured urinary bladder. The animal presented with anuria for 8 days **(A)**; abdominocentesis revealed bloody uroperitoneum, with blood urea nitrogen 172 mg/dl and serum creatinine 22 mg/dl. **(B)** Longitudinal abdominal scan using a 3.5 MHz sector probe showing massive uroperitoneum (UP) with the right kidney (RK) and fibrinous material (F) floating in the anechoic fluid. **(C)** Transverse abdominal scan revealing the left kidney (LK) and spleen (S) floating within the UP; intestines and liver were also visualized suspended in the fluid. Transrectal ultrasonography further confirmed massive UP in the pelvic cavity with visualization of the ruptured urinary bladder.

**Figure 14:**
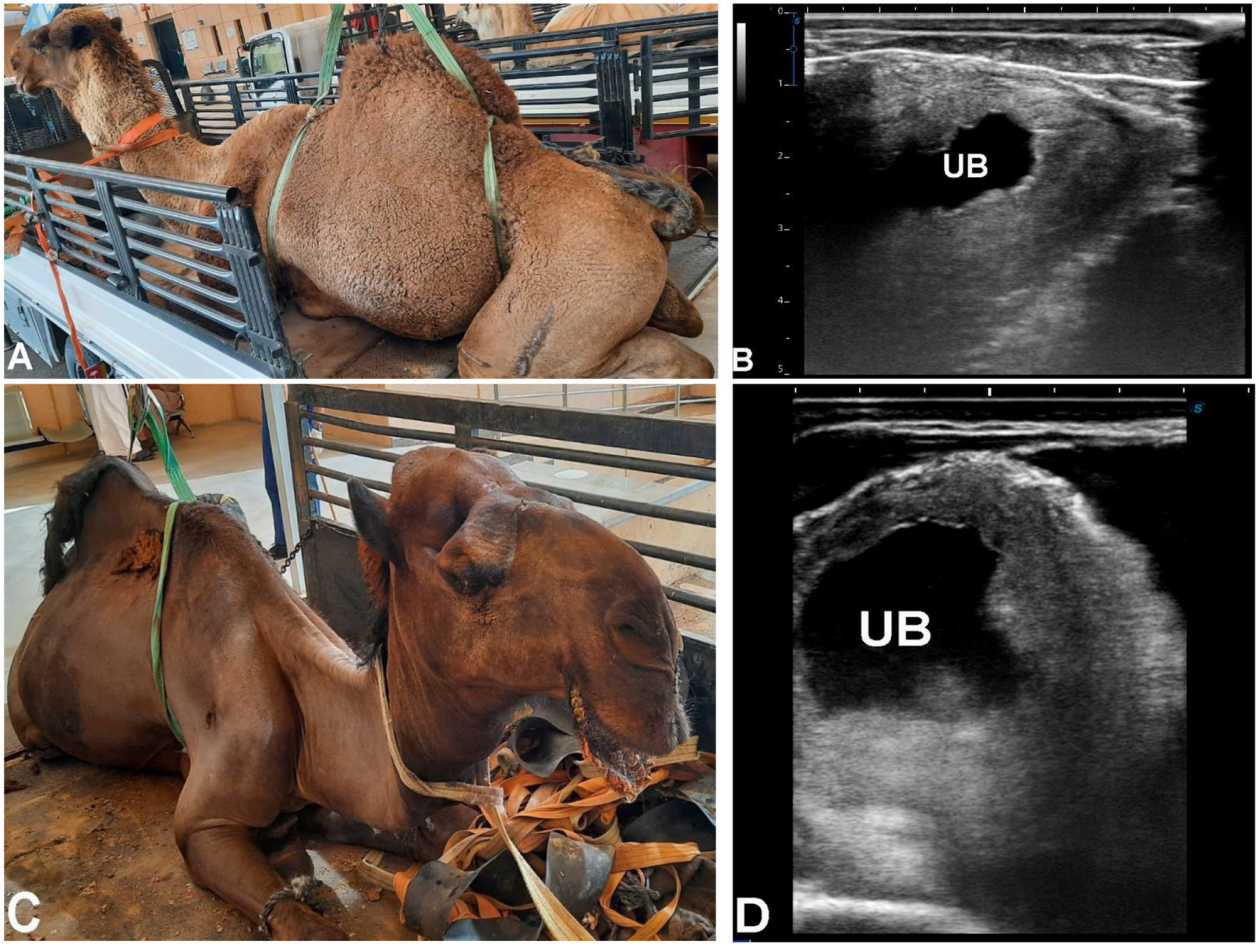
Ultrasonographic evaluation of ruptured urinary bladder in two male dromedary camels. Camel in **(A)** presented with severe impaction and anuria for 10 days, and camel in **(C)** with anuria for 10 days. Both had previously received diuretics. Blood urea nitrogen levels were 169 mg/dl and 185 mg/dl, and serum creatinine levels were 19.5 mg/dl and 17.5 mg/dl, respectively. Abdominal ultrasonography using a 3.5 MHz sector probe revealed massive uroperitoneum (UP) with floating intestines, liver, and kidneys. Transrectal ultrasonography **(B, D)** confirmed ruptured urinary bladder within the pelvic cavity, showing a collapsed bladder with markedly thickened wall.

**Figure 15:**
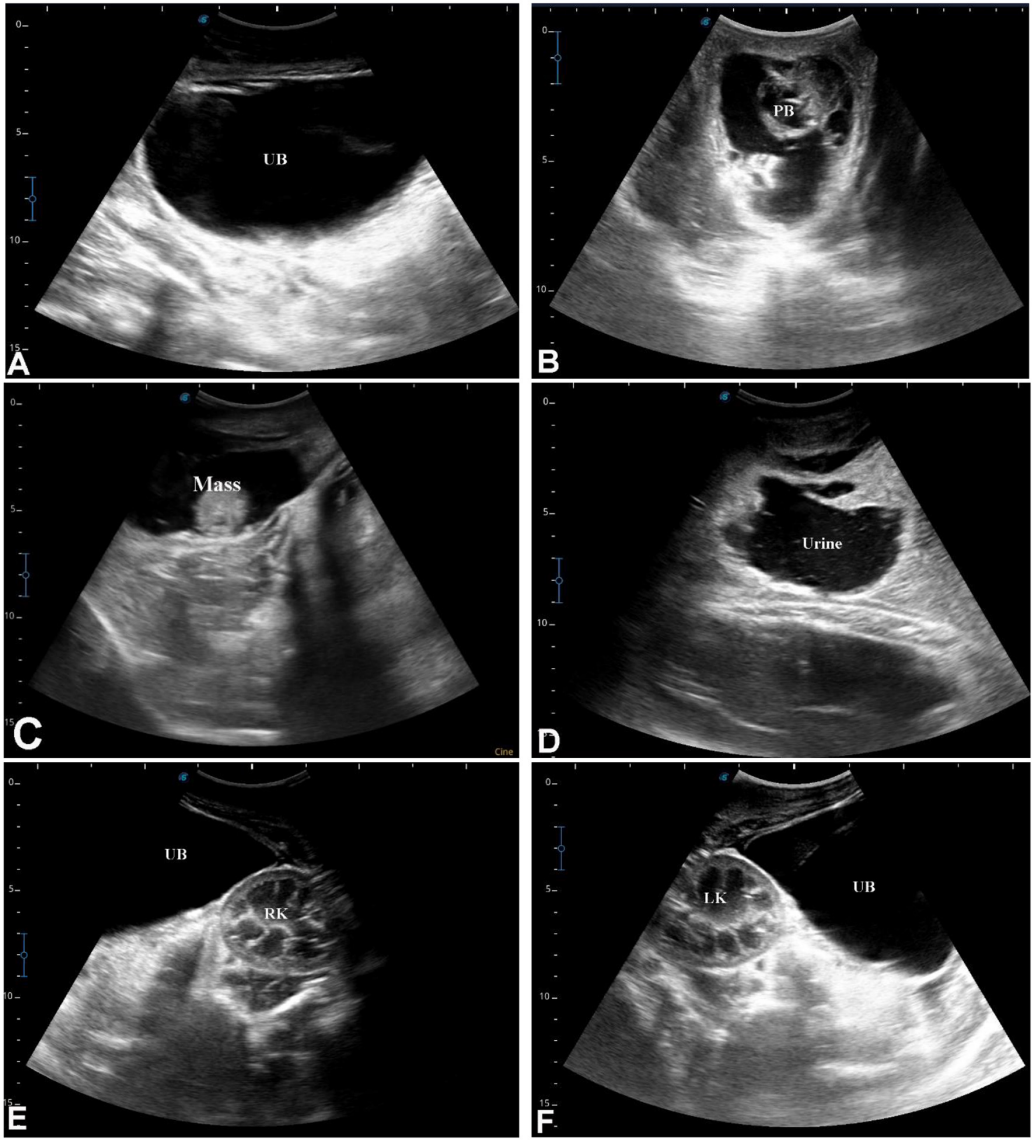
Ultrasonographic evaluation of ruptured urethra in a 3-month-old male dromedary camel calf. Transrectal and transabdominal ultrasonography were performed using a 5.0 MHz linear probe. **(A)** shows the intact urinary bladder (UB). **(B)** demonstrates the ruptured penile body (PB). **(C)** depicts a partially obstructing mass within the penile lumen, while **(D)** shows urine infiltrating the surrounding soft tissues. **(E, F)** display the intact urinary bladder (UB) adjacent to the right kidney (RK) and left kidney (LK), illustrating the relationship of the bladder to the renal structures and confirming normal kidney positioning.

Seventy-three camels were included, classified based on sonographic evidence of urine leakage or traumatic disruption of the urinary tract. Sixty camels (all males, aged 3–20 years) presented with abdominal pain, anuria, urine dribbling, and progressive abdominal enlargement. Ultrasonography revealed large volumes of hypoechoic peritoneal fluid with floating abdominal organs, including intestines, liver, kidneys, and spleen, findings consistent with uroperitoneum (Figures 12, 13). Transrectal ultrasonography identified a collapsed urinary bladder with a markedly thickened wall, freely floating within abdominal fluid, consistent with bladder rupture (Figure 14). Thirteen camels [7 females (53.8%), 6 males (46.2%), aged 3 months−19 years] showed sonographic evidence of an intact but distended urinary bladder with hyperechoic intraluminal material, along with gross enlargement of the penile body and extravasated urine surrounding the urethra. An obstructing intraluminal object was visualized within the penile urethra (Figure 15), consistent with traumatic or foreign body–associated urethral obstruction.

### 3.6 Group 6 (G6 – histopathologically confirmed neoplastic lesions)

Two female camels presented with gradual weight loss and intermittent abdominal pain. Ultrasonography revealed well-defined to irregular renal masses with variable echogenicity. In both cases, ultrasound-guided biopsy or aspiration was performed, and histopathological examination confirmed renal cell carcinoma in both cases.

## 4 Discussion

This retrospective observational study describes ultrasonographic patterns associated with urinary tract–related clinical syndromes in 1,100 dromedary camels examined over a 14-year period, representing one of the largest datasets currently available in camelid urology. Given the retrospective design and inconsistent availability of laboratory and histopathological data, this study is descriptive and does not aim to establish definitive etiologic diagnoses or validate ultrasonography against laboratory gold standards. Consequently, descriptive statistics (raw frequencies and proportions) were used, and inferential analyses were not performed.

All diagnostic categories discussed below represent clinically suspected syndromes primarily defined by sonographic appearance, with supplementary clinical information and confirmatory testing when available. The findings emphasize ultrasonography as a frontline, pattern-recognition tool in real-world clinical settings where advanced diagnostics are often unavailable. The chronicity, diversity, and overlap of sonographic findings highlight the need for cautious interpretation, transparent terminology, and diagnostic integration.

### 4.1 Group 1 (G1 – disorders presenting with grossly discolored urine and intraluminal echogenic bladder content)

Camels in this group were classified based on visibly discolored urine and ultrasonographic evidence of intraluminal echogenic material or bladder wall abnormalities. In the absence of systematic urinalysis, these cases reflect disorders presenting with urine discoloration rather than confirmed hemorrhagic disease, as alternative causes (hemoglobinuria, myoglobinuria, pigmenturia) cannot be excluded.

The wide range of sonographic findings—including echogenic intravesical material, bladder wall irregularity, renal pelvic dilation, cavitary renal lesions, and free abdominal fluid—illustrates the multifactorial nature of urine discoloration syndromes. Structures interpreted as blood clots may also represent sediment, inflammatory debris, or mass-like lesions. Concurrent renal abnormalities highlight overlap between bladder- and kidney-associated disease. Despite these limitations, ultrasonography remains clinically valuable for localizing lesions, identifying concurrent abnormalities, and guiding diagnostics such as aspiration or biopsy. The findings align with previous camelid reports (27, 28) while reinforcing cautious terminology.

### 4.2 Group 2 (G2 – disorders characterized by bladder wall thickening, echogenic intraluminal material, or impaired bladder emptying)

This group encompasses camels classified primarily on the basis of characteristic bladder- and lower urinary tract–related sonographic findings, rather than confirmed inflammatory or infectious etiologies. Bladder wall thickening, corrugation, and echogenic intraluminal material are non-specific and may reflect inflammation, infection, crystalluria, or early obstruction. The absence of urinalysis or culture in most cases precludes differentiation between these causes.

In male camels with balanitis, ultrasonography primarily demonstrated secondary urinary outflow dysfunction, including bladder distension and pelvic urethral dilation (32). Similarly, cases identified as urinary bladder paralysis were based on marked bladder distension with intact walls and intraluminal echogenic material, without evidence of mechanical obstruction. Lack of neurologic, metabolic, or laboratory confirmation requires interpretation as sonographically apparent bladder atony rather than confirmed neurogenic disease.

### 4.3 Group 3 (G3 – renal cavitary or heterogeneous lesions consistent with abscessation)

Renal cavitary and heterogeneous parenchymal lesions constitute a substantial subset, with microbiological confirmation available in some cases via ultrasound-guided aspiration (*Staphylococcus lugdunensis*, S. *aureus*). Lesions lacking microbiological confirmation should be interpreted as sonographically consistent with abscessation rather than confirmed abscesses, as similar appearances may reflect necrosis, chronic hematomas, or other focal pathologies. Ultrasonography provides reliable lesion localization, assessment of size and laterality, and guidance for aspiration, supporting its central role in suspected renal infection.

### 4.4 Group 4 (G4 – obstructive disorders)

Obstructive disorders are among the most reliably defined by ultrasonography, due to characteristic acoustic features of uroliths and predictable anatomic consequences. Predominantly affecting males, findings such as echogenic foci with distal acoustic shadowing, bladder distension, urethral dilation, and renal pelvic enlargement are considered diagnostic of obstruction, and laboratory confirmation is not strictly required. Despite incomplete dietary and metabolic data, these sonographic patterns remain highly applicable in clinical practice (5, 29).

### 4.5 Group 5 (G5 – traumatic and urine leakage–associated disorders)

Traumatic urinary tract disorders exhibited distinctive sonographic features, particularly in uroperitoneum and bladder rupture (33). Hypoechoic peritoneal fluid with floating viscera is diagnostic of urine leakage, and transrectal ultrasonography effectively identified bladder collapse and rupture sites. Outcome data were insufficient to correlate severity with prognosis, but ultrasonography proved rapid, non-invasive, and reliable in emergency presentations.

### 4.6 Group 6 (G6 – histopathologically confirmed neoplastic lesions)

Urinary tract neoplasia was rare (30). Inclusion was restricted to histopathologically confirmed cases, avoiding reliance on ultrasonography alone. While sonographic features were documented, ultrasound was not considered diagnostic of neoplasia without tissue confirmation. The small number of cases precludes generalizations regarding prevalence or tumor behavior, but provides reference images and reinforces the need for biopsy or aspiration when mass lesions are detected (34, 35).

### 4.7 Research strengths and limitations

The study's strengths include a large sample size, long observation period, and consistent ultrasonographic methodology performed by the same clinical team using standardized protocols. Limitations are summarized as follows: the retrospective design, inconsistent availability of laboratory, microbiological, neurologic, and histopathological data, incomplete treatment and follow-up documentation, and lack of uniform image archiving. These limitations mean that many diagnostic labels represent clinically suspected syndromes rather than confirmed diseases, with inevitable overlap between categories. They are not methodological failures but reflect real-world clinical practice, where ultrasonography often serves as the primary diagnostic modality.

## 5 Conclusion

This long-term retrospective study demonstrates that ultrasonography is a practical, accessible, and highly informative tool for characterizing urinary tract–related clinical syndromes in dromedary camels. Across 1,100 cases over 14 years, ultrasound consistently identified structural abnormalities of the kidneys, bladder, urethra, and peritoneal cavity, enabling lesion localization, assessment of disease extent, and guidance for targeted diagnostic procedures. Although many findings could not be definitively linked to specific etiologies due to incomplete laboratory confirmation, this sonographic-first approach accurately reflects real-world clinical practice. The dataset provides a valuable descriptive foundation to support improved recognition, diagnostic reasoning, and management of urinary tract disorders in camelids, while highlighting the need for future prospective studies integrating laboratory diagnostics, histopathology, and follow-up outcomes.

## Data Availability

The original contributions presented in the study are included in the article/supplementary material, further inquiries can be directed to the corresponding author.
